# The Relationship between New Stroke Onset and Serum Thiocyanate as an Indicator to Cigarette Smoking

**DOI:** 10.2188/jea.11.233

**Published:** 2007-11-30

**Authors:** Hongbing Wang, Michikazu Sekine, Hiroshi Yokokawa, Shimako Hamanishi, Xiaoli Chen, Michio Sayama, Yuchi Naruse, Hideaki Nakagawa, Sadanobu Kagamimori

**Affiliations:** 1 Department of Welfare Promotion and Epidemiology, Toyama Medical and Pharmaceutical University, Toyama, Japan.; 2 Faculty of Engineering, Toyama University, Toyama, Japan.; 3 Department of Community and Geriatric Nursing, Toyama Medical and Pharmaceutical University, Toyama, Japan.; 4 Department of Public Health, Kanazawa Medical University, Ishikawa, Japan.

**Keywords:** serum thiocyanate, stroke, cigarette smoking, biomarker

## Abstract

The case subjects were 67 stroke patients (27 males and 40 females, mean age 65.7±7.1), who suffered from stroke attack and had participated in annual medical check-up between 1987 and 1988 at Oyabe Public Health Center. The controls, which were adjusted for sex, age and blood pressure level with the case subjects, were selected from participants attending their annual medical check-up in 1987-1988. The serum thiocyanate level of case subjects were significantly higher than that of controls (p<0.05). The usefulness of measurement of serum thiocyanate level as an indicator of smoking status was assessed by using multiple logistic regression analysis adjusting for body mass index, mean blood pressure, and serum cholesterol was found. An odds ratio of 3.00 (95% confidence interval: 1.06-8.48, p<0.05) in cerebral infarction.

It is considered that serum thiocyanate may be useful as an indicator of smoking status to assess the relationship with stroke onset, especially for cerebral infarction.

## INTRODUCTION

Smoking habits has been identified as a risk factor for stroke in several studies^1^^-^^3^^)^. Wolf et al.^3^^)^ reported that cigarette smoking was a significant risk factor for stroke, especially cerebral infarction, and that there was a dose-response relationship between the number of cigarettes smoked per day and the relative risk of stroke. However, these results were not confirmed in other studies^4^^-^^6^^)^. The potential of misclassification in assessing the smoking status with self-assessment reports may be one of the factors leading to these different results. Therefore, it is difficult to evaluate smoking status by using self-administered questionnaire only, and the objective method should be used to assess smoking status.

Serum thiocyanate concentration is one of the objective indicators for assessing smoking habits. The validity of serum thiocyanate as an indicator of cigarette smoking has been studied by several investigators^7^^, ^^8^^)^. The relatively long half-life (about 1-2 weeks) of serum thiocyanate makes its measurement particularly suitable for evaluating habitual smoking.

There were few investigations on the direct relationship between serum thiocyanate level and stroke onset. We investigated the relation of serum thiocyanate with references to other associated risk factors of stroke using a nested case-control design in a community-based cohort study.

## MATERIALS AND METHODS

A nested case-control study was designed and carried out based on the data from the Oyabe Community-based Stroke Registry, which was started from 1966. The hospitals and ambulance stations in and around the Oyabe district, Toyama Prefecture, Japan had been required to provide information on stroke patients. All residents aged 40 years or above (6,954 persons, including 2,421 males and 4,533 females) in the studied area were involved in an annual medical check-up between 1987 and 1988.

In this study, case group included 67 stroke patients who had been recorded in the Oyabe Community-based Stroke Registry at Oyabe Public Health Center.. The cases participated in their annual medical check-up between 1987 and 1988, and subsequently suffered from stroke attacks in a 4-year follow-up period from 1988 to 1991. The diagnosis of stroke was made with reference to the reported physician’s diagnosis and other relevant information following the 9th revision of the International Classification of Diseases. The detail of the diagnosis and confirmation was described elsewhere^9^^)^. The subjects were 27 males and 40 females, and their average age was 65.7±7.1 years. The stroke subtypes were determined according to death certificate and confirmed by the method described previously^9^^)^. Of the all stroke patients, 43 patients were classified into cerebral infarction, 13 patients intracerebral hemorrhage, 7 patients subarachnoid hemorrhage, and the rest were unknown origin.

Subjects in control group (67 persons), matched for sex, age and blood pressure level with the cases, were selected from participants whose serum samples had been stored during annual medical check-up in 1987 to 1988. The value of height, weight, blood pressure, alcohol consumption, and concentration of total serum cholesterol was read from their annual medical check-ups for both case and control subjects. According to the self-reported smoking habits each subject was classified into subgroups of non-smokers, light smokers who smoke 10 cigarettes or less per day, moderate smokers who smoke 11-20 cigarettes per day, and heavy smokers with more than 20 cigarettes per day. The body mass index (BMI) (weight in kg divided by height squared in meters, kg/m^2^) and mean blood pressure (MBP = (2DBP + SBP)/3) were also calculated.

Serum thiocyanate was measured by the modified method of Butts et al^10^^)^. using frozen serum samples. The serum samples of both cases and controls were collected at the annual medical check-up in 1987 to 1988, and froze for future measurement. A volume of 0.8-1.0 ml serum sample was diluted to 2ml with saline and 1 ml of NaClO_4_ (0.1M) added and shaken, then 1 ml of trichloroacetic acid (30% W/V) was added and shaken vigorously. The mixture was stored statically at room temperature for 20 minutes, subsequently was centrifuged for removal protein-precipitate. One milliliter of ferric nitrate [Fe(NO_3_)_3_•9H_2_O] (10 g/l) was added into 2ml of the clear supernatant, and shaken vigorously. Thereafter, absorbance at 455 nm of the samples was measured immediately. The quantitative of thiocyanate was done along with standard curve made by taking KSCN (50-200 *μ*mol/l solution) as standard of SCN^−^ running through the same operation program as serum samples.

The protocol of this study was explained to the participants in detail before collecting the needed private information and blood samples. The informed consents were obtained from all cases and controls. Between case and control groups, differences in mean values of age, BMI, MBP, serum total cholesterol at the time of medical check-up between 1987 and 1988 were examined by paired t-test. The distribution of serum thiocyanate concentration did not follow a normal distribution. Then, the values of serum thiocyanate were logarithmically transformed to become normal distribution. The ratio of smokers to non-smokers and ratio of persons who intake alcohol daily to those who don’t intake alcohol were examined by chi-squared test. The multiple logistic regression was used to examine the effect of serum thiocyanate and other factors on the stroke incidence, with the results showed as odds ratio (95% confidence interval). The sample size of this study was not large enough to permit further analysis by stratifying some factors such as sex and age groups. All statistical analyses were performed on a SPSS 7.5.1J software package. A p-value of less than 0.05 was considered significant.

## RESULTS

The general characteristics of those participating in the study are shown in Table 1. Among female cases, both all strokes and cerebral infarction had a higher mean level of serum total cholesterol than controls (all strokes 205.9 mg/dl vs. controls 178.6 mg/dl, cerebral infarction 212.9 mg/dl vs. controls 173.7 mg/dl). Age, BMI, and MBP showed no significant difference between patients and controls. For both all strokes and cerebral infarction, the ratio of persons who intake alcohol diurnally in control group was significantly higher than that in patient group. There were no difference with the ratios of smokers to non-smokers between stroke patients and controls, but male stroke patients had a significantly higher mean level of serum thiocyanate than control (stroke cases 72.4 *μ*mol/l, controls 48.7 *μ*mol/l, p<0.05) (Table 2).

**Table 1.** General characteristics of stroke patients and controls.

	All strokes				Cerebral infarction			
	
	Males		Females		Males		Females	
			
	Patients (n=27)	Controls (n=27)	Patients (n=40)	Controls (n=40)	Patients (n=21)	Controls (n=21)	Patients (n=22)	Controls (n=22)
Age (years)	66.1±7.1	65.3±6.5	65.4±7.1	64.8±7.3	67.1±7.3	66.0±6.8	67.7±5.8	66.9±5.8
BMI (kg/m^2^)	22.5±2.8	22.4±2.4	23.2±3.1	23.1±2.9	22.5±2.7	22.1±2.5	23.3±2.5	23.2±2.9
MBP (mmHg)	102.3±12.5	100.3±12.6	99.8±15.8	95.0±15.1	101.5±13.1	100.3±11.8	95.8±11.9	93.2±13.1
T-Chol (mg/dl)	186.5±50.6	181.2±51.4	205.9±47.2*	178.6±60.9	188.8±56.7	173.7±48.2	212.9±32.2*	173.7±64.9
Smoking (%) ^@^	48.1	55.6	0	0	52.4	57.1	0	0
Alcohol (%) ^§^	22.4*	55.6	2.5	0	19.0*	52.4	4.8	0

BMI, body mass index ; MBP, mean blood pressure ; T-Chol, serum total cholesterol ;

^@^ : smoking(%) ; ratio of smokers

^§^ : alcohol(%) ; ratio of persons who have alcohol intake every day

* p<0.05, patients vs controls

**Table 2.** Serum thiocyanate level (*μ*mol/l) of stroke patients and controls.

	Patients			Controls		
	
	No.	Meam	SD	No.	Meam	SD
All strokes						
Males	27	72.4*	1.7	27	48.7	2.4
Females	40	50.8	1.5	40	42.8	1.8
Cerebral infarction						
Males	21	69.9*	1.7	21	54.1	2.0
Females	22	49.0	1.5	22	46.8	1.6

The paired t-test was conducted between cases and controls with logarithmically transformed values of serum thiocyanate, and then the results changed back ;

* P<0.05, patients vs controls

The contribution of age, sex, BMI, MBP, total serum cholesterol, serum thiocyanate and alcohol intake on susceptibility to stroke attack was assessed using multiple logistic regression. The risk of cerebral infarction stroke was significantly increased for high serum thiocyanate concentration with an odds ratio of 3.00 (95% confidence interval, 1.06 to 8.48), and for high serum total cholesterol with an odds ratio of 3.03 (1.14 to 8.10), and significantly decreased for alcohol intake with an odds ratio of 0.22 (0.05 to 0.99) (Table 3). Furthermore, it was analyzed using multiple logistic regression with the parameter replacing the smoking status by questionnaire for serum thiocyanate. There was no significant change of odds ratio by smoking status (Table 3).

**Table 3.** Analysis of the risk of cerebral infarction using multiple logistic regression.

	univariate analysis		multivariate analysis	
	
	Odds ratio	(95% CI)	Odds ratio	(95% CI)
Age	1.69	(0.56 - 5.06)	1.85	(0.60 - 5.71)
Sex	2.27	(0.72 - 7.22)	2.13	(0.72 - 6.35)
BMI	1.29	(0.46 - 3.59)	1.24	(0.72 - 6.35)
MBP	0.87	(0.26 - 2.96)	0.74	(0.21 - 2.62)
T-Chol	2.77	(1.08 - 7.10)*	3.03	(1.14 - 8.10)*
Smoking				
<=10	0.65	(0.10 - 4.29)		
>10	0.77	(0.13 - 4.42)		
Thiocyanate			3.00	(1.06 - 8.48)*
Alcohol	0.37	(0.08 - 1.63)	0.22	(0.05 - 0.99)*

CI: confidence interval, Age: age>=65 years vs age<65 years,

Sex: male vs female,

BMI: body mass index; BMI>=24 vs BMI<24 kg/m^2^,

MBP: mean blood pressure ; MBP>=110 vs MBP<110 mm Hg,

T-Chol: serum total cholesterol; T-Chol>=200 vs T-Chol<200mg/dl,

Thiocyanate: serum thiocyanate; continuous valuable with logarithm transformation

Smoking: self-reported smoking status; smoking number <=10/day,

>10/day vs not smoking

Alcohol: daily vs not daily

* p<0.05

## DISCUSSION

In epidemiological studies on cigarette smoking, self-assessment reports and direct interviews have usually been taken as an indicator of smoking status. However, the misclassification due to these methods is inevitable, because, for example, individuals who sometimes mistake their cigarette consumption and smokers who have failed and/or want to stop smoking are apt to underreport. In addition to actual cigarette consumption, smoking status varies with depth of inhalation, frequency of puffing and proportion of each cigarette consumed. Therefore, it is important that we make an effort to gain an objective marker of smoking status.

To assess smoking status objectively, carbon monoxide in expired air, blood carboxyhemoglobin (COHb), cotinine or thiocyanate has been measured^11^^)^. Among these indicators, expired carbon monoxide can be easily measured and blood carboxyhemoglobin measurement has been substituted by expired air carbon monoxide measurements. Because of short half-life of expired carbon monoxide (about four hours)^12^^)^, it can’t be used to assess smoking habit correctly. Cotinine, metabolite of nicotine, is specific to cigarette smoking and is one of the best indicators to distinguish smokers from non-smokers, but it also has a short half-life (30 hours).

Thiocyanate is a metabolite of serum hydrogen cyanide derived from cigarette smoking and has a relatively long half-life (about 1-2 weeks). Serum thiocyanate is suitable for evaluating smoking habits, although it is not specific to cigarette smoking^13^^-^^15^^)^. Serum levels of thiocyanate among non-smokers and light to heavy smokers have been reported by several investigators. Foss et al^13^^)^. reported that the mean serum thiocyanate level in non-smokers was 33.9 *μ*mol/l for males and 33.5 *μ*mol/l for males. In moderate smokers (15-19 cigarettes per day), the mean level was 76.3 *μ*mol/l for males, and in heavy smokers (more than 25 cigarettes per day), the mean level was 87.3 *μ*mol/l for males. In a study on a Japanese rural population, Kasagi et al.^15^^)^ reported that the optimal cut-off point was 50 *μ*mol/l for differentiating smokers from non-smokers,.

The proportion of cerebral infarction among all strokes has reached about 70% in recent years. As a design of case-control study nested in a community-based cohort study, the subjects in this report were the entire new onset of stroke patients up to present data analysis since the annual check-up in 1987-1988, and the information about the subjects was collected prospectively. The proportions of various types of stroke are similar to the distribution in the total population. In this report, we observed that serum thiocyanate level in stroke patient was significantly higher than that in controls. The results were identical for all stroke patients and for cerebral infarction patients.

Cigarette smoking has been established as a risk factor for ischaemic heart diseases for many years but only in the past decade has its role as a risk factor for stroke and its pathological subtypes been delineated^1^^)^. Because stroke is pathologically and aetiologically heterogeneous, it is likely that the association between smoking and stroke risk is even stronger for certain pathological and aetiological subtypes of stroke, and weaker for others. For the relatively small sample size in this study, we have to focus on analyzing the data of subtype of cerebral infarction. The results showed very different statistical relationship between smoking and the risk of stroke, with self-reported number of cigarette smoked per day or measured serum thiocyanate used as the indicator of smoking status respectively. The risk of cerebral infarction elevated significantly with increment in the logarithmically transformed concentration of serum thiocyanate. On the other hand, with self-reported number of cigarettes instead of the serum thiocyanate in the same multiple logistic regression model, there is no statistical significant effect of smoking on the risk of cerebral infarction any more. Some points have been proposed to explain this kind of discrepancy. Studies using biochemical markers of smoking to check volunteered information suggest that self-reporting may be unreliable^16^^)^. Self-reports of smoking have showed a marked degree of digit preference, with the vast majority of smokers reporting in multiples of 10 cigarettes per day. Then, self-reports of number of cigarettes per day may be biased towards round numbers, rather than the exact number of cigarettes smoked per day^17^^)^. The results reported by Daly RJ, et al^18^^)^ indicated that use of serum thiocyanate levels revealed a considerably high level of smoking denial by smokers in ischaemic heart diseases (55%) and in peripheral vascular diseases (28%). It is almost impossible to estimate the effect of cigarette smoking on health correctly with such high proportion of smoking denial by using the method of self-reporting. The questionnaire information including the item of smoking and others has been rechecked, but the actual cigarette smoking behavior of individual subjects in this study cannot be reconfirmed at present. It seems plausible that the stroke risk of cigarettes smoking may be estimated much more precisely with serum thiocyanate than with self-reported number of cigarettes smoked per day as the indicator of smoking status, considering the small sample size in this study and relative weaker stroke risk to smoking.

High blood pressure is the most important of the known risk factors for all stroke subtypes^19^^, ^^20^^)^, although we did not determined it in our study since it was limited as one of the match factors for selecting control subjects. In our study, high serum total cholesterol increased the risk of cerebral about 3-fold, and was consistent with the result of other studies^21^^-^^24^^)^, although the relationship between serum total cholesterol and the risk for stroke attack is not so clear. Two recently published papers in the same issue of a neurological journal gave completely inconsistent answers^25^^, ^^26^^)^. However, there is also mounting evidence that serum total cholesterol concentration is inversely associated with the risk of hemorrhagic stroke, and, in contrast, high serum total cholesterol concentration seems to increase the risk of cerebral infarction^21^^-^^23^^)^. Heavy alcohol consumption might result in an increased stroke risk. However, in a recent study by Sacco et al.^27^^)^, earlier observations of a protective effect of moderate alcohol consumption on ischaemic stroke risk have been confirmed, and this kind of effect was independent of gender, race, current smoking, hypertension and other disease status. We also observed the similar effect of alcohol consumption on cerebral infarction.

Finally, it is considered that the measurement of serum thiocyanate as indicator of smoking status may be more precisely and useful to assess the risk of stroke attack than the self-reported number of cigarettes smoked per day. In this study, we could not analyze the data of intracerebral hemorrhage and other types of stroke patients because of the small sample sizes. Well-designed study should be conducted to determine the reason why there is so big different result between the self-reported smoking status and measured serum thiocyanate used as the indicator of smoking habit, and find the way to solve the problem in the future.
